# Individual differences in frequency and impact of daily memory lapses: results from a national lifespan sample

**DOI:** 10.1186/s12877-023-04363-6

**Published:** 2023-10-17

**Authors:** Jacqueline Mogle, Jennifer R. Turner, Sakshi Bhargava, Robert S. Stawski, David M. Almeida, Nikki L. Hill

**Affiliations:** 1 Department of Psychology, Clemson University, 418 Brackett Hall, Clemson, SC 29634 USA; 2 Department of Psychology, University of Hawaii at Hilo, Hilo, HI USA; 3 Department of Patient Centered Outcomes Assessment, RTI-Health Solutions, Research Triangle Park, NC USA; 4 Institute of Public Health and Wellbeing, University of Essex, Colchester, UK; 5 College of Health and Human Development, The Pennsylvania State University, University Park, PA USA; 6 Ross and Carol Nese College of Nursing, The Pennsylvania State University, University Park, PA USA

**Keywords:** Daily memory, Subjective memory, Daily diary, Impacts of memory

## Abstract

**Background:**

Everyday memory problems are believed to increase with age, leading many researchers to focus on older ages when examining reports of memory lapses. However, real world memory lapses are ubiquitous across the adult lifespan, though less is known about the types of problems and their impacts at younger ages. The current study examined occurrence and impacts of memory lapses using daily diaries in a broad age range and whether characteristics of lapses varied across age, gender, or education level.

**Methods:**

Using an 8-day daily diary protocol, 2,018 individuals (ages 25–91) provided reports of their experiences of two types of daily memory lapses (retrospective and prospective) as well as the impact those lapses had on their emotional and functional well-being that day. Using multilevel modeling, we examined the likelihood of reporting memory lapses and their impacts on daily life and whether these depended on age, gender, or education level.

**Results:**

Participants reported lapses on approximately 40% of days; retrospective memory lapses were significantly more likely than prospective lapses. Older ages and higher education level were related to greater likelihood of reporting retrospective lapses. Women (compared to men) were more likely to report prospective memory lapses. Women also tended to report greater impacts of their memory lapses. Lower education levels were related to greater impacts of memory lapses compared to higher education levels. Interestingly, age was not related to impacts of lapses.

**Discussion:**

Our results indicate that memory lapses are common across the lifespan and that those individuals more likely to report lapses are not necessarily those that experience the greatest impacts of those lapses on daily life. Additional work is needed to understand the daily experience of memory lapses and how they differentially affect individuals regardless of age, gender, and education.

**Conclusions:**

Memory lapses are an important aspect of daily life across the lifespan and require measurement in an individual’s real-world environments. Better measurement of these experiences will allow the development of more sensitive measures of changes in cognitive functioning that may impact an individual’s ability to live independently.

## Introduction

Research on everyday cognitive difficulties tends to focus on older adults’ experiences given age-related decline in performance-based assessments of memory and the importance of memory to functional independence. However, memory is required to meet a variety of cognitive demands in daily life regardless of age, and failure to meet these demands can result in a range of challenges across the lifespan [1]. For example, forgetting to take a medication can have substantive health consequences at any age. Understanding memory lapses as they occur in the real world in a lifespan sample would allow the examination of the types of lapses experienced and how individuals appraise their emotional and functional impacts. Further, knowing *who* is most likely to experience different types of daily memory lapses and whether the impacts of lapses differ based on other individual difference characteristics such as gender or education would provide insight into the need for group-specific interventions for daily memory functioning. The current study examines data from a large national sample of adults ages 25 to 91 to explore the characteristics of daily memory lapses and these characteristics differ based on the age, gender, or education level of the reporter.

Reports of cognitive difficulties associated with memory are frequently examined among older adults. This is in response to a critical need to better understand normative changes in everyday memory functioning with age as well as to identify the subtle early declines associated with non-normative aging trajectories (e.g., mild cognitive impairment or Alzheimer’s disease [2, 3]. To capture these subjective declines, older adults are frequently asked to provide a rating of how often they experience memory problems across weeks, months, or even years in a range of everyday tasks (e.g. *Memory Functioning Questionnaire* [4], *Prospective and Retrospective Memory Questionnaire* [PRMQ] [5]). Although informative about general tendencies in different types of memory difficulties among older adults, there is a need to explore the characteristics and impacts of specific memory experiences [6]. Further, age-related decrements in performance-based assessments of memory are well-established [7], self-reports of everyday memory problems are stable across the lifespan [8]. Broadening the assessment of experiences of memory problems beyond general tendencies and among individuals at younger ages would expand our understanding of everyday memory problems and the key characteristics of memory problems that have clinical and research importance.

One important point of differentiation is among types of everyday memory problems. In both performance-based assessments and self-report questionnaires researchers attempt to discriminate among prospective memory (i.e., memory of future intentions) and retrospective memory (i.e., memory for past information [9]). Due to the number of tasks individuals need to accomplish in daily life, researchers have hypothesized that problems with prospective memory will be more common and have greater impacts on everyday functioning relative to retrospective memory [9]. Although some questionnaires separate these two types (e.g., PRMQ [5]), a frequent goal is to create a summary of the total number of problems (e.g., Cognitive Change Index [10]). This is particularly relevant as there is a disconnect among lab-based and real-world performance on prospective memory tasks. Older adults tend to perform similarly to younger adults in real-world prospective memory, despite showing deficits in lab-based tasks [11] suggesting that prospective memory problems in real-world situations deserve additional attention [12].

Complicating the role of age in reporting on everyday memory problems are individual characteristics such as gender and education. Women are more likely to report memory problems compared to men regardless of the type of memory problem [5, 13], though gender differences in performance-based tasks tend to be more mixed [14]. In contrast, education differences in reports of everyday memory problems remain unclear [15, 16], despite long-standing observations of lower education as a risk for poorer memory performance across different types of memory [17, 18]. The emphasis of existing questionnaires on general tendencies and long-term recall of everyday memory problems potentially obscures existing real-world differences in the occurrence and impact of memory problems among these different subgroups.

Daily diary approaches provide a novel window into the everyday memory experiences of adults across the lifespan. Asking about recent real-world experiences increases the verisimilitude (i.e., real world applicability) of a report by gathering details about a specific memory lapse. This is particularly useful when the experience of interest is believed to occur repeatedly and/or over a brief window of time (e.g., several times a week [19, 20]) as is the case with memory lapses [21]. Critically, assessing specific experiences of forgetting also allows identification of characteristics that discriminate across similar occurrences [22]. The collection of specific characteristics would enable pinpointing those memory problems that are most relevant for intervention targeting [23]. For example, forgetting to attend a book club meeting and forgetting to attend a doctor’s appointment can have different impacts on health and well-being. However, even among different types of doctor’s appointments, forgetting to attend a routine check-up differs from forgetting to attend a follow-up to a medical procedure. Experiential assessments such as daily diaries can provide the information needed to potentially discriminate these types of forgetting experiences (cf., [1]) through obtaining appraisals of specific experiences [24].

Examining appraisals of the emotional and functional impacts of daily experiences of forgetting as well as individual differences in these appraisals would expand on the importance of lapses as a daily experience. As noted above, certain types of lapses may be more strongly associated with substantial personal impacts relative to other types [9]. In a recent descriptive exploration of daily memory lapses, we established that the most frequent lapses were not those appraised as having the greatest emotional and functional impacts [25]. As one example, forgetting where something was placed occurred on approximately 12% of days but was rated lower in terms of impacts relative to less frequent lapses such as forgetting to attend an appointment. However, this work focused on a sample of adults over the age of 50 and whether these trends hold at younger ages remains unclear. Further, given the known differences in appraisals of other daily experiences among men and women (e.g., stress [26]; pain [27]) and individuals with differing levels of education (e.g., stress, [28]) there is a need to understand how individual characteristics relate to reports of impact for daily memory lapses.

## Current study

The overall goal of the current study was to examine the occurrence and appraisals of different types of memory lapses in a national sample of adults across the adult lifespan. We also examined whether/how occurrence or appraisals of lapses varied by age, gender, education, or their interactions. We examined differences due to age given extensive work indicating decrements in objective memory performance beginning as early as 30 years of age [29, 30], however, evidence suggests older adults report fewer everyday memory problems compared to younger (e.g., PRMQ [5]). Based on this previous work, we hypothesized that older adults would report fewer daily memory lapses and appraise lapses as less impactful relative to younger and middle-aged adults. We considered gender differences due to work indicating that women (relative to men) tend to report greater numbers of everyday memory problems [31] and hypothesized that women in our sample would also tend to report more daily memory lapses and appraise lapses as having more impactful relative to men. Finally, differences in daily memory lapse experiences due to education was an exploratory analysis. Some previous work shows that individuals with lower levels of education report greater numbers of everyday memory problems [32, 33], though these effects are mixed. Overall, this study aimed to determine the extent to which findings from previous work using conventional reports of everyday memory problems is congruent with reported experiences of daily memory lapses.

## Methods

### Participants

We conducted a secondary data analysis of two cohorts of the National Institute on Aging (NIA) funded *Midlife in the United States* (MIDUS; http://midus.wisc.edu/) study: the third wave of the MIDUS study (MIDUS-3; collected between 2013–2019) and the MIDUS Refresher (MIDUS-R; collected between 2011–2014) sample. The MIDUS study is a longitudinal survey collected approximately every ten years that examines the lives of Americans living in the United States using a large representative sample and measures family, life, and leisure characteristics, as well as health functioning and well-being. Importantly, a random subset of individuals was selected to complete a daily diary portion over an 8-day period to report on daily stress, affect, memory lapses, and social interactions. The MIDUS Refresher sample included 782 participants and the MIDUS-3 sample included 1,236 participants, leading to a total sample of 2,018 participants for the present analyses (see Table 1 for all demographic characteristics). Given that the daily diary collection protocol was the same between samples, we combined these datasets into one to examine our research questions of interest. The final sample included individuals across the adult lifespan with 8% between the ages of 25 and 35 (*n* = 166), 12% between 36 and 45 (*n* = 245), 25% between 46 and 55 (*n* = 515), 25% between 56 and 65 (*n* = 506), 22% between 66 and 75, and 7% over the age of 76.

**Table 1:** Participant demographic characteristics

Characteristics of Interest	MIDUS-3 (*n* = 1,236)	MIDUS-R (*n* = 782)	Combined MIDUS (*N* = 2,018)
Age (in years; *M*, *SD*)	*M* = 62.62, *SD* = 10.33	*M* = 47.91, *SD* = 12.67	*M* = 56.92, *SD* = 13.37
Gender, *n* (%)			
Women	707 (57.2%)	435 (55.6%)	1142 (56.6%)
Men	529 (42.8%)	347 (44.4%)	876 (43.4%)
Race, *n* (%)			
White	1027 (83.1%)	659 (84.3%)	1686 (83.5%)
Black	37 (3.0%)	50 (6.4%)	87 (4.3%)
Native American or American Eskimo	6 (0.5%)	11 (1.4%)	17 (0.8%)
Asian or Pacific Islander	4 (0.3%)	7 (0.9%)	11 (0.5%)
Other	23 (1.9%)	51 (6.5%)	74 (3.7%)
Did not disclose	139 (11.2%)	4 (0.5%)	143 (7.1%)
Education, *n* (%)			
Completed High School or less	306 (24.8%)	160 (20.5%)	466 (23.1%)
Some College	321 (26.0%)	232 (29.7%)	553 (27.4%)
College Degree or beyond	494 (40.0%)	390 (49.9%)	884 (43.8%)
Did not disclose	115 (9.3%)	0 (0.0%)	115 (5.7%)
Income, *n* (%)			
< $25,000	526 (42.6%)	232 (29.7%)	758 (37.6%)
$25,000 – $49,999	360 (29.1%)	183 (23.4%)	543 (26.9%
$50,000 – $74,999	117 (9.5%)	125 (16.0%)	242 (12.0%)
$75,000 – $99,999	43 (3.5%)	68 (8.7%)	111 (5.5%)
> $100,000	34 (2.8%)	93 (11.9%)	127 (6.3%)
Did not disclose	156 (12.6%)	81 (10.4%)	237 (11.7%)

The combined sample includes the third wave of MIDUS (MIDUS-3) as well as the Refresher sample (MIDUS-R). The age range in MIDUS-3 was 43–91 years and the age range in MIDUS-R was 25–75 years

*Note*. *MIDUS* Midlife in the United States Study

All participants completed the same daily diary protocol. Participants received a phone call each evening for 8 consecutive evenings. During this call, that lasted approximately 20 min, a trained interviewer asked a series of questions about their daily experiences. All questions were asked in the same order each evening and participants received monetary compensation for their participation in the study.

Participants were included in the present study if they had completed at least one daily diary survey and were not missing the demographic variables of gender, age and education, given that these were the primary targets of interest. For the total sample, there were 16,144 possible daily assessments (2,018 participants × 8 days of assessments) and 15,150 were successfully completed, leading to an overall compliance of 93.8%. The average number of surveys completed by participants was 7.51 (*SD* = 1.59; range 1–8), suggesting overall high compliance.

## Measures

### Demographics

Participants reported their age, gender, and education. Age is scored as the number of years since birth at the time of the MIDUS data collection. Gender was collected as a binary variable and coded as 1 for women and 0 for men. Education was coded as high school or less = 0, some college = 1, and Bachelor’s degree or beyond = 2 consistent with previous work using this variable [34].

### Daily Memory Lapse Checklist (DMLC)

Replicating Mogle and colleagues [21, 25], our primary instrument of assessment was the DMLC. The MIDUS form of this checklist contains 9 items that pertain to retrospective (i.e., memory for previously learned information) memory lapses and prospective (i.e., memory for future behaviors and activities) memory lapses. Items on the retrospective subscale includes forgetting someone’s name, where something was placed, a word during a conversation, and important information. Items on the prospective subscale include forgetting an errand or chore, to take a medication, why you entered a room, to finish a task, or to attend a meeting or appointment. For both subscales, the total number of lapses were summed to create a composite number of prospective (range 0–5) and retrospective (range 0–4) daily memory lapses. Daily reliability^1^ for prospective lapses was 0.70 and for retrospective lapses it was 0.69.

### Appraisals of memory lapses

When participants reported experiencing a memory lapse, they were prompted to indicate the level of perceived negative impact on two domains: irritation (*How much did forgetting these things bother you?*) and interference (*How much did forgetting these things interfere with your routine today?*). Both follow-up measures of perceived consequences were reported on a 0 (*Not at all*) to 10 (*Very much*) point analog scale. These questions were asked for both the prospective and retrospective items, leading to prospective irritation (*M*_reliability_ = 0.82) and interference (*M*_reliability_ = 0.75) and a retrospective irritation (*M*_reliability_ = 0.86) and interference (*M*_reliability_ = 0.79) outcome measures.

## Analytic plan

Analyses were completed in a series of steps. First, items were scored consistent with previous work with this scale [25]. Using these scores, we examined the number of days with any memory lapse reported, total number of days of a given type of memory lapse, average ratings of irritation and interference for each type, as well as the frequency of specific memory lapses within each type—including which lapses were appraised as most irritating and interfering across persons. Comparisons of frequencies of total lapses across types were made using Wilcoxon Signed Ranks tests with specific Friedman test contrasts to compare frequencies of lapses to account for the count nature of these variables. Comparisons of irritation and interference were made using paired-samples *t*-tests for total scores and repeated-measures mixed models for individual lapse ratings as these were continuous.

To address our substantive questions about differences across age, gender, and education, substantive analyses using multilevel modeling (MLM) examined group differences in the frequency of reporting memory lapses or the perceived irritation and interference associated with a daily memory lapse. MLM was appropriate for the present analyses as it accounts for the nesting of days within persons as well as unequal numbers of observations for participants [35, 36]. Multilevel Poisson regression (SAS *proc glimmix*) was utilized for frequency of prospective and retrospective lapses and linear multilevel regression (SAS *proc mixed*) was used for ratings of irritation and interference. When significant main effects of age, gender, or education were found, we estimated simple effects by group or 1 standard deviation difference on age to determine the size of the effect using a standardized difference (*d* = mean difference divided by the estimated standard deviation).

### Covariates

To control for other potential person- and day-level variables that could influence the reporting of memory lapses and their consequences, the covariates of participant dataset, race, and daily stress were included in the MLM analyses. Dataset (MIDUS-3 = 1; MIDUS-R = 2) was included to control for potential cohort differences between participant groups based on possible differences in assessment period or historical effects, given the wide range of data collection (i.e., years 2011–2019). Race was recoded into a dummy variable corresponding to non-Hispanic, White (1) or Other (0), and daily stress was a binary variable indicating if the person reported experiencing a stressor (e.g., argument [37]) on that day.

## Results

### Descriptive analyses 

Participants reported memory lapses on 40.9% of assessments (see Table 2). Prospective memory lapses occurred on 22.2% of assessments (*N*_days_ = 3,226) and retrospective memory lapses occurred on 30.2% (*N*_days_ = 4,386); this difference was significant (*Z* = 20.24, *p* < 0.001). On approximately 11.4% of assessments, participants reported experiencing both retrospective and prospective memory lapses on the same prompt (*N*_days_ = 1,653). The most common retrospective lapse was forgetting *where something was placed* (15.0% of days; Friedman χ^2^ (3) = 1451.40, *p* < 0.001), and the most common prospective lapse was forgetting *why they entered a room* (9.4% of days; Friedman χ^2^(4) = 852.81, *p* < 0.001). Participants were more likely to report experiencing multiple retrospective memory lapses (e.g., *forgetting someone’s name* and *forgetting a word*) at one measurement occasion compared to prospective lapses (*t* (1953) = 13.61, *p* < 0.001, 95% CIs [0.11, 0.15]).

**Table 2:** Daily frequency and descriptive analyses for the daily memory lapse checklist

			**Irritation (1 – 10)**		**Interference (1 – 10)**	
	***N***_**days**_	**% of Total Days**	***M* (*SD*)**	**Range**	***M* (*SD)***	**Range**
**Prospective Memory**	3,226	22.2%	2.87 (2.28)	1–10	1.77 (2.78)	1–10
*To do an errand/chore*	958	6.6%	3.31 (2.46)	1–10	2.00 (1.85)	1–10
*To take medication on time*	687	4.7%	3.05 (2.41)	1–10	1.87 (1.69)	1–10
*To attend a meeting/appointment*	293	1.9%	3.73 (2.72)	1–10	2.31 (2.22)	1–10
*Why you entered a room*	1,363	9.4%	2.79 (2.32)	1–10	1.78 (1.62)	1–10
*Finish something*	886	6.1%	3.33 (2.50)	1–10	2.23 (2.02)	1–10
Two or more Prospective Complaints (Range 2—5)	770	5.3%	3.69 (2.64)	1–10	2.42 (2.09)	1–10
**Retrospective Memory**	4,382	30.2%	2.96 (2.33)	1–10	1.69 (1.55)	1–10
*Someone’s name*	1,717	11.8%	2.88 (2.28)	1–10	1.59 (1.50)	1–10
*Where something was placed*	2,183	15.0%	3.28 (2.52)	1–10	2.04 (1.87)	1–10
*A word during a conversation*	1,777	12.2%	3.02 (2.35)	1–10	1.71 (1.56)	1–10
*Something you wanted to remember*	446	3.1%	4.76 (2.77)	1–10	2.95 (2.53)	1–10
Two or more retrospective complaints (Range 2—4)	1,321	9.1%	3.49 (2.54)	1–10	2.09 (1.92)	1–10

Total number of days = 15,150

For retrospective memory lapses, 25.6% of participants (*n* = 517) never reported a lapse, while 4.6% participants (*n* = 92) reported at least one memory lapse every day. Among prospective memory lapses, 34.1% of participants (*n* = 689) never reported experiencing a memory lapse over the 8 days, while 2.9% participants (*n* = 58) reported experiencing at least one memory lapse every day. A subset of participants never reported experiencing memory lapses of either type throughout the 8 days (*n* = 322; 16.0%), while 1.6% (*n* = 32) always reported both types of lapses.

On average, at the person-level, retrospective memory lapses were rated 2.93 (*SD* = 1.98) on irritation and 1.67 (*SD* = 1.22) on interference; prospective memory lapses were rated as 2.70 (*SD* = 1.81) on irritation and 1.66 (*SD* = 1.24) on interference. The difference between lapse types was significant for irritation, *t* (1071) = 4.35, *p* < 0.001, 95% CI of the difference [0.13, 0.34], d = 0.12, though not for interference, *p* = 0.69 (d = 0.008).

Examining specific experiences of retrospective memory lapses, forgetting *something you wanted to remember* was rated highest on irritation (*M* = 4.76, *SD* = 2.77; *p* < 0.001) and interference (*M* = 2.95, *SD* = 2.53; *p* < 0.001). For prospective memory lapses, forgetting *to attend a meeting or appointment* was rated as both the most irritating (*M* = 3.73, *SD* = 2.72; *p* < 0.001) and the most interfering (*M* = 2.31, *SD* = 2.22; *p* = 0.013). In contrast, forgetting *someone’s name* was rated lowest on both measures of impact for retrospective lapses (*M*_irritation_ = 2.88, *SD* = 2.28; *M*_interference_ = 1.59, *SD* = 1.50; both *p*s < 0.001) and forgetting *why you entered a room* was rated lowest for prospective lapses (*M*_irritation_ = 2.79, *SD* = 2.32, *p* < 0.001; *M*_interference_ = 1.78, *SD* = 1.62, *p* = 0.081). Finally, when participants reported experiencing two or more prospective or retrospective memory lapses at the same assessment, this was associated with elevated levels of interference and irritation.

### Demographic comparisons

Below we present findings from models that examined whether prospective and retrospective memory lapses and their impacts (i.e., irritation and interference) differ by age, gender, and education, or their interactions after accounting for dataset, daily stress, and race. All models initially tested three-way interactions; however, none of these interactions were significant (all *p*s > 0.096). Therefore, models only including two-way interactions are presented.

#### Frequency of retrospective and prospective memory lapses

For retrospective lapses, older age was related to slightly greater odds of reporting a lapse (*OR* = 1.09, 95% CI: 1.023–1.169, *p* = 0.009). Having at least a college degree was also related to greater odds of reporting a retrospective lapse compared to both other groups (high school or less: *OR* = 1.31, 95% CI: 1.13–1.52, *p* = 0.001; Some college: *OR* = 1.29, 95% CI: 1.13–1.48, *p* = 0.0002). The main effect of gender was not significant (*p* = 0.087).

For prospective lapses, women were more likely to report lapses than men (*OR* = 1.365; 95% CI: 1.19–1.56, *p* < 0.001), but age and education were not significantly associated with number of prospective lapses (*p*s < 0.114). No interaction effects were significant for either type of memory lapse (all *p*s > 0.13).

#### Emotional impacts due to retrospective and prospective memory lapses

For retrospective lapses, women rated lapses as more irritating compared to men (*b* = 0.37, *SE* = 0.11, *p* = 0.001, *d* = 0.25). Individuals with the lowest levels of education rated lapses more irritating relative to individuals with the highest levels of education (*b* = 0.35, *SE* = 0.14, *p* = 0.01, *d* = 0.24). Age was not related to irritation ratings for retrospective memory lapses (*p* = 0.39).

For prospective lapses, older age was related to higher irritation (*b* = 0.12, *SE* = 0.06, *p* = 0.05, *d* = 0.15). The main effects of gender and education on irritation ratings for prospective memory lapses were qualified by a significant interaction (*p* = 0.047). This interaction indicated that women in the lowest education category (completed high school or less) had higher levels of irritation relative to women with some college experience (*b* = 0.542, *SE* = 0.18, *p* = 0.003, *d* = 0.42) and women with a college degree or beyond (*b* = 0.723, *SE* = 0.17, *p* < 0.001, *d* = 0.56); this was not true for men across levels of education (all *p*s > 0.16). Women who completed high school or less also rated their prospective lapses as higher in irritation compared to men with the same level of education (*b* = 0.767, *SE* = 0.243, *p* = 0.002, *d* = 0.59).

#### Functional impacts due to retrospective and prospective lapses

For retrospective lapses, interference ratings were higher among women relative to men (*b* = 0.134, *SE* = 0.064, *p* = 0.036, *d* = 0.18). Main effects of age (*p* = 0.146) and education (*p* = 0.144) as well as the two-way interactions (*p*s > 0.40) were not significant.

For prospective lapses, interference ratings were highest among individuals who completed high school or less and these ratings were significantly higher than individuals with a college degree or beyond (*b* = 0.226, *SE* = 0.093, *p* = 0.015, *d* = 0.31). Main effects of age (*p* = 0.499) and gender (*p* = 0.109), and all the two-way interactions (all *p*s > 0.105) were not significant.

## Discussion

The current study examined a daily measure of memory lapses in a national lifespan sample of adults to understand the types of lapses experienced and how they were appraised as impacting daily functioning. Consistent with our previous work in adults ages 50 and older, we found that retrospective memory lapses are reported on about one third of days while prospective memory lapses occur on a quarter of days [21, 25]. Retrospective memory lapses were more frequent than prospective lapses but were equivalent in their rated emotional and functional impact. With respect to demographic variations among daily memory lapses, older adults (compared with younger) tended to report more retrospective lapses, but not significantly different levels of irritation and interference stemming from the lapses. In contrast, when prospective lapses occurred, older adults reported greater irritation (though not interference) compared to younger adults. Women also reported more frequent prospective memory lapses, and appraised lapses as more emotionally and functionally impactful, compared with men. Finally, individuals with higher levels of education reported more frequent retrospective lapses, but those with lower levels of education appraised both types of lapses as having a greater impact on daily life.

The finding that retrospective memory lapses occur more often than prospective memory lapses is consistent with previous work using daily measures of memory lapses [21, 25]. Although prospective memory demands are hypothesized to be more common in daily life [9], we found that the two types of lapses were comparable in their appraised interference in daily life. Although retrospective lapses were appraised as significantly more irritating, the difference between lapse types was small (about a quarter point difference on a 10-point scale). This is in contrast to our previous work indicating that prospective lapses are associated with greater perceptions of future consequences [21]. This may be in part due to the reporting method; some impacts of prospective memory lapses may not have been realized or occurred at the time of the survey.

In contrast to previous work using self-reports of memory functioning, older adults reported more retrospective memory lapses and appraised prospective lapses as more impactful compared to younger adults in the current sample [21, 38]. This finding is consistent with evidence from performance-based tasks as older adults are more likely to be experiencing declines in memory performance. One reason for the inconsistency of our findings with previous work on self-reports of memory problems is the separation of prospective and retrospective reports. The age differences in frequency were specific to retrospective lapses while no age differences were found for prospective memory lapses. The general lack of age differences in the impacts of memory lapses (i.e., no differences in interference and only differences in irritation around prospective lapses) may reflect a bias in reporting: older adults may be more likely to downplay their memory lapses as a self-protective coping mechanism as they age [39].

Differences in daily memory lapse occurrence and impact due to gender are an addition to the current literature. Women reporting greater numbers of prospective memory lapses regardless of age suggests that differences in gender roles could influence the number of to-be-engaged-in activities, and therefore the number of opportunities for lapses. For example, time use surveys indicate women remain responsible for a larger portion of household duties in addition to paid work [40], as well as greater perceived busyness across the lifespan [41]. This difference in role responsibilities could reflect greater opportunities for noticeable lapses in daily life as well as greater impact of lapses that do occur.

The findings related to education differences in frequency and impact of memory lapses are similar to patterns identified in previous work around daily stress [34, 42]. Individuals lower in education reported fewer lapses; however, their appraised impact on daily functioning was greater. This is similar to work that finds individuals with higher levels of education report more daily hassles (e.g., arguments) but appraise these events as lower in stressfulness [43]. This may indicate that individuals with more education have more opportunities for lapses due to differences in daily activities (e.g., work activities) but are better able to cope with the consequences of those lapses due to differences in available resources (e.g., financial income).

### Limitations

The current study demonstrates the types of daily memory lapses as well as their appraised impact among a large adult lifespan sample, however there are several limitations that should be noted. First, while the MIDUS study utilizes a national sample of the United States, the present sample was relatively homogenous regarding race and ethnicity, with over four-fifths of respondents identifying as White-only. This limited our ability to examine potential demographic differences in memory lapses related to racial or ethnic identification. It is possible that specific types of memory lapses may be more or less salient (including the strength of daily impacts related to memory lapses) among different racial or ethnic groups. A second limitation stems from the frequency of assessment of the daily memory lapses. The design of the daily assessment in MIDUS (once-a-day for 8 days) permitted us to capture the relative frequency of memory lapses and provided evidence of the commonality of these experiences (i.e., approximately 41% of assessments had at least one memory lapse), however assessing memory lapses at only one time point per day leaves room for reporting errors when it comes to lapses. For example, lapses characterized by low (or no) irritation and/or interference might not be noticed or recalled during the interview and major lapses might not be noticed until an external reminder appears (e.g., when a person misses an appointment and does not realize until they receive a bill). Additionally, reports could be biased by an individual’s memory ability. Those individuals with poorer memory (e.g., older adults) may be less likely to recall experiences with forgetting, rendering our counts of lapses as an undercount for those individuals. A final limitation relates to the brevity of the present checklist, which likely does not capture all possible types of memory lapses that individuals experience in their daily lives. For example, forgetting “to finish something” or “something you wanted to remember” were appraised as relatively more impactful than other types of lapses, yet not knowing what *something* referenced limits the interpretation of this item. An important future direction would be to incorporate fill-in-the-blank or “other” responses to create more customized types of lapses, with a goal of further improving this measure.

## Conclusions

Memory is important for navigating daily life and healthy functioning. Experiencing a memory lapse is colloquially associated with increased age, despite past empirical work finding that memory lapses occur among individuals of all ages [21, 38]. Other individual difference factors, like gender or education, potentially influence the frequency or impact of memory lapses, but few studies have specifically focused on these relationships particularly in daily life. The current study addressed this gap by utilizing a measure of prospective and retrospective daily memory lapses in a national study of adults across the lifespan, which included the additional key components of the appraised daily impacts (irritation and interference) related to memory lapses. Memory lapses occur frequently, but the results of this study suggest that not all memory lapses have equal impact on daily life, and that the likelihood of reporting a memory lapse (or not having a lapse) depends on gender, education, as well as the type of memory lapse. Identifying those who are potentially more at risk for having worse outcomes stemming from memory lapses (in conjunction with frequency) is a first step towards supporting better daily memory functioning across the lifespan.

## Data Availability

Data for the Midlife in the United States study is available at the Inter-university Consortium for Political and Social Research at the University of Michigan, project numbers: ICPSR 36346; ICPSR 36532. The direct accessible link of source can be found here. The point of contact is author, Dr. Jacqueline Mogle.

## Footnotes

1 Daily, repeated-measures reliability for the checklist and memory lapse consequences were calculated using guidelines from Hox et al. [35, 36] using intraclass correlations (ICCs) and the average number of completed assessments (i.e., 7).
